# COVID-19 Vaccine During Pregnancy and Perinatal Outcomes

**DOI:** 10.7759/cureus.33240

**Published:** 2023-01-02

**Authors:** Amani Almutairi, Mufareh Asiri, Salem Alsuwaidan, Reem Sufyani, Shumukh AlSalem, Joud Alghamdi

**Affiliations:** 1 Pharmaceutical Care Services, King Saud Medical City, Riyadh, SAU; 2 Obstetrics and Gynecology, King Saud Medical City, Riyadh, SAU; 3 Diabetes and Endocrinology, King Saud Medical City, Riyadh, SAU; 4 Medical Education and Simulation, King Saud Medical City, Riyadh, SAU

**Keywords:** perinatal outcome, birth weight, covid 19 in pregnancy, coronavirus disease 2019 (covid-19), covid-19 vaccine

## Abstract

Introduction

When COVID-19 vaccination started, there was little data on the safety of immunization against COVID-19 infection in pregnant women. Previous studies revealed no safety concerns for pregnant women or newborns who received a messenger ribonucleic acid (mRNA) COVID-19 vaccine during pregnancy. This study aimed to investigate the effects of COVID-19 vaccination on pregnant women and on perinatal outcomes.

Methods

This cross-sectional study was conducted in a maternity hospital in King Saud Medical City. It started in January 2022 and ended in June 2022. The questionnaire was developed and validated by experts. This study included all women admitted to the postpartum ward who were more than 18 years old and had received the COVID-19 vaccine. The study excluded women who had no proof of their vaccination status or who could not complete the questionnaire. The primary outcome was the effect of COVID-19 vaccination on gestational age and birth weight. The secondary outcomes included the development of polyhydramnios, oligohydramnios, mode of delivery, Apgar score, postpartum hemorrhage, and neonatal intensive care unit admission.

Results

A total of 365 pregnant women participated in this study. The mean gestational age of the unvaccinated women was 38.83 ± 1.62 weeks, which was significantly (p < 0.001) higher than that of vaccinated women (37.69 ± 2.9 weeks). In addition, the average birth weight for the unvaccinated women was 2.96 ± 0.4 kg, which did not differ significantly (p = 0.89) from that of vaccinated women (2.97 ± 0.66 kg).

Conclusion

COVID-19 vaccination, regardless of the type of vaccine received before, during, or after pregnancy, is not associated with any unfavorable perinatal outcomes for pregnant women or neonates.

## Introduction

COVID-19 was considered a devastating worldwide pandemic after it caused the deaths of millions of people worldwide [1]. Many types of research have studied the effects of COVID-19 on physical and mental health during pregnancy [2-4 ]. Compared to nonpregnant women of the same reproductive age, pregnant women are at higher risk of severe illness and mortality from COVID-19, along with an increased risk of unfavorable pregnancy outcomes such as preterm birth [5-7]. At the onset of vaccination during this pandemic, there was a paucity of safety data on COVID-19 immunization of pregnant women. However, over time the available evidence has documented no harmful maternal or neonatal consequences of the administration of COVID-19 vaccines to pregnant women, and there is also a growing body of evidence that supports the safety of such vaccinations [8-12]. Findings from three safety monitoring systems revealed no safety concerns, either for pregnant women who received a messenger ribonucleic acid (mRNA) COVID-19 vaccine in their late pregnancy or for their newborns (NBs) [13]. The acceptance of vaccination among pregnant women varies, even with the increasing evidence. However, the immunization rate is still low among pregnant women [14]. The Pfizer and Moderna vaccines are the preferred vaccines for eligible pregnant women of any age because healthcare workers have more extensive experience with their use in pregnancy [15]. Therefore, this study was conducted to investigate the effect of different COVID-19 vaccines approved in Saudi Arabia on perinatal outcomes.

## Materials and methods

This cross-sectional study was conducted in a maternity hospital in King Saud Medical City (KSMC) from January 2022 to June 2022 after being approved by the KSMC’s institutional review board, with reference number H1RI-06-Feb22-01. The questionnaire was developed and validated by experts. A pilot study was conducted before starting the official study to test the validity of the questionnaire, and the pilot study participants were excluded from this study. All participants gave their written informed consent before they were included in the study. Each participant’s vaccination status was checked using the Tawakkalna (Saudi Data and Artificial Intelligence Authority (SDAIA), SAU) mobile application, which showed the vaccination status (number of doses, dates, and type of vaccines). This application is mandatory for all citizens, whether Saudi or non-Saudi. In this study, all women admitted to the postpartum ward who were more than 18 years old and who had received the COVID-19 vaccine (Pfizer-BioNTech, Moderna, or Oxford-AstraZeneca) either before or during pregnancy were included as one arm and compared to unvaccinated women as a second arm. We excluded women who either did not have proof of their vaccination status or who refused to complete the questionnaire.

The questionnaire was divided into two parts; the first part contains the participants’ demographic data such as their age, height, previous obstetrical history, smoking habits (smoker, ex-smoker, or non-smoker), and immunization status against COVID-19, including the number of COVID-19 vaccine doses they had already received and whether they were unvaccinated. Furthermore, the type of COVID-19 vaccine received (Pfizer, Moderna, or AstraZeneca) and the pregnancy trimesters when the COVID-19 vaccine doses were received were reported. The second part of the questionnaire contains the pregnant women’s medical history, such as the gestational age at delivery, chronic diseases, or any health conditions, including preeclampsia, premature birth, or gestational diabetes. Pregnancy-related hypertension illnesses, oligohydramnios, polyhydramnios, or amniotic fluid stained with meconium were other pregnancy issues that were investigated. In addition, delivery and postpartum were studied including the types of delivery (vaginal, vacuum, or cesarean) or any placental abruption that may have occurred during labor as well as postpartum hemorrhage or fever. Moreover, NB characteristics were recorded, such as birth weight, Apgar score, and neonatal intensive care unit (NICU) admission. After the sample was completed, the participants were divided into two groups, vaccinated and unvaccinated women, and thereafter the data was analyzed to show the differences. The primary outcome was the effect of the COVID-19 vaccine on gestational age and birth weight. The secondary outcomes included the development of polyhydramnios, oligohydramnios, mode of delivery, Apgar score, postpartum hemorrhage, and NICU admission. The mother and the NB were followed up until the time of discharge from the hospital. Data were collected, cleaned, and verified in an Excel sheet, after which they were coded and analyzed using Statistical Product and Service Solutions (SPSS) (IBM SPSS Statistics for Windows, Version 26.0, Armonk, NY). Chi-square and analysis of variance (ANOVA) tests were used to compare the variables to each other.

## Results

Data were collected by professionals from the postpartum ward. A total of 365 women filled out the questionnaire. The mean body mass index was 28.6 ± 5.1kg/m^2^, and the modal age range was 26-35 years. A total of 289 participants had taken the first dose of the COVID-19 vaccine, 266 had taken the second dose, and 102 had taken the third dose. Approximately 57% of the subjects had COVID-19 previously, either before or during pregnancy. It was noticed that some subjects had become infected with COVID-19 after vaccination, with 88 (24%) during pregnancy and 116 (32%) after delivery. We found that 32 women had gestational diabetes, 26 had hypertension, and 33 had preeclampsia.

Regarding amniotic fluid changes, it was noticed that 14 participants had oligohydramnios and 14 had polyhydramnios. It was found that 197 women had a vaginal delivery, 166 underwent cesarean delivery, and only two NBs were delivered by vacuum. It was also found that 14 participants had placenta abruption and 26 experienced postpartum hemorrhage. These complications affected the length of hospitalization because it was found that 184 women were hospitalized for one day, 141 for two to three days, and 40 for more than three days, as shown in Table 1.

**Table 1:** Comparisons of the characteristics of vaccinated and unvaccinated women. *P-value = <0.05 None of the NBs was delivered with a positive COVID-19 test. NB = newborn; 95% CI: 95% confidence interval; NICU: neonatal intensive care unit

Women’s related diseases	Unvaccinated (n = 76) No. & (%)	Vaccinated (n = 289) No. & (%)	Chi-Square	
			P-value	95% CI
Mode of age (26-35)	34 (44.7%)	149 (51.6%)	0.2851	(-5.65%) to 18.98%
Hypertension	1 (1.3%)	24 (8.3%)	0.0317*	0.65% to 10.90%
Thyroid disorder	4 (5.3%)	17 (5.9%)	0.8421	(-7.22%) to 5.23%
Preeclampsia	1 (1.3%)	32 (11.1%)	0.0082*	3.24% to 14.08%
History of premature birth	4 (5.3%)	64 (22.1%)	0.0008*	8.09% to 22.85%
COVID-19 before vaccination	13 (17.1%)	111 (38.4%)	0.0005*	9.935 to 30.2%
COVID-19 before pregnancy	9 (11.8%)	62 (21.5%)	0.0578	(-0.42%) to 17.17%
COVID-19 during pregnancy	10 (13.2%)	69 (23.9%)	0.0442*	0.25% to 18.56%
Gestational diabetes mellitus	4 (5.3%)	28 (9.7%)	0.2285	(-6.39%) to 3.41%
Oligohydramnios	0 (0%)	14 (4.8%)	0.0518	(-3.65%) to 9.49%
Polyhydramnios	1 (1.3%)	13 (4.5%)	0.1967	(-0.38%) to 7.91%
Meconium amniotic fluid	4 (5.3%)	25 (8.7%)	0.3314	(-2.84%) to 6.42%
Congenital anomalies	1 (1.3%)	6 (2.1%)	0.6527	(-4.59%) to 8.38%
Cesarean delivery	39 (51.3%)	127 (43.9%)	0.2496	(-5.06%) to 3.41%
Vacuum delivery	0 (0%)	2 (0.7%)	0.4651	(-5.04%) to 19.65%
Vaginal delivery	37 (48.7%)	160 (55.4%)	0.2977	(-4.13%) to 2.50%
Placental abruption	4 (5.3%)	10 (3.5%)	0.47	(-5.74%) to 18.96%
Postpartum hemorrhage	8 (10.5%)	18 (6.2%)	0.1945	(-2.46%) to 9.47%
1-day hospitalization	44 (57.9%)	140 (48.4%)	0.141	(-5.08%) to 5.12%
2-3 hospitalization	24 (31.6%)	117 (40.5%)	0.1568	(-3.10%) to 21.41%
>3 hospitalization	8 (10.5%)	32 (11.1%)	0.8818	(-3.51%) to 19.87%
5 minutes Apgar score >7	72 (94.7%)	276 (95.5%)	0.7689	(-8.82%) to 7.16%
5 minutes Apgar score ˂7	0 (0%)	10 (3.5%)	0.0986	(-3.62%) to 8.53%
I don’t know	4 (5.3%)	3 (1%)	0.0146*	(-1.56%) to 6.30%
NB respiratory depression	0 (0%)	14 (4.8%)	0.0518	0.54% to 11.84%
NB fever	0 (0%)	2 (0.7%)	0.4651	(-0.38%) to 7.91%
NB admitted NICU	1 (1.3%)	23 (8%)	0.0366*	(-4.13%) to 2.5%

The main finding results showed that the mean gestational age for the unvaccinated women was 38.83 ± 1.62 weeks, which is considered significantly higher (p = 0.001) than that for vaccinated women (37.69 ± 2.9 weeks). However, the average gestational age of vaccinated women with the second and third doses was similar to that of vaccinated women with the first dose (37.63 weeks and 37.89 weeks, respectively).

Corresponding results showed that the mean birth weight for unvaccinated women was 2.96 ± 0.4 kg, which did not differ significantly (with p = 0.89) from the mean birth weight for vaccinated women (2.97 ± 0.66 kg). It increased after the second and third doses to 2.98 ± 0.68 kg and 3.11 ± 0.56 kg, respectively. None of these variations were statistically significant, as shown in Tables 2 and 3.

**Table 2:** Mean gestational age and birth weight for vaccinated and unvaccinated women.

Vaccinated vs. unvaccinated	Unvaccinated (N = 76)		Vaccinated (N = 289)		P-value
	Mean	St. Dev	Mean	St. Dev	
Gestational age	38.83	1.62	37.69	2.865	0.001*
Birth weight (Kg)	2.9566	0.39609	2.9679	0.66365	0.8873

**Table 3:** Differences in the mean gestational age and birth weight according to the number of vaccine doses.

Number of COVID-19 vaccine doses received		1st dose (n = 289)		2nd dose (n = 266)		3rd dose (n = 102)		P-value
		Mean	St. Dev	Mean	St. Dev	Mean	St. Dev	
Gestational age	37.69		2.865	37.63	2.926	37.89	2.911	0.742149
Birth weight (Kg)	2.9679		0.66365	2.9751	0.6774	3.1118	0.56258	0.137462

**Figure 1:**
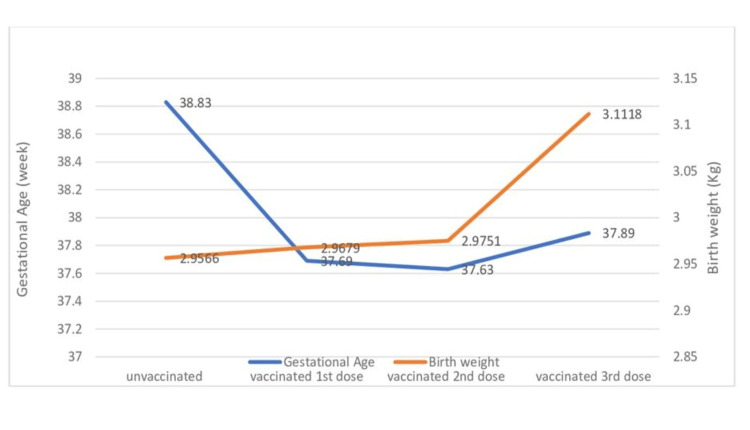
Gestational age in weeks and birth weight for NBs after delivery for vaccinated and unvaccinated women. The figure also shows differences between vaccinated women with different doses. NBs: new borns

A comparison of the gestational age at delivery and birth weight between vaccinated and unvaccinated delivered women is shown in Figure 1, together with data for the second and third doses.

Conversely, regarding the perinatal outcome for NBs, we found that 14 NBs had respiratory complications, two had a fever, and 24 NBs were admitted to the NICU, but we noticed that none tested positive for COVID-19 irrespective of whether the mothers were vaccinated or unvaccinated.

## Discussion

The Saudi government offered free COVID-19 vaccines to all clients, irrespective of whether they were Saudi or non-Saudi citizens. The Saudi Minister of Health approved Pfizer-BioNTech, Moderna, Oxford-AstraZeneca, and Johnson & Johnson COVID-19 vaccines; however, the participants of this study received all types of vaccines except for the Johnson & Johnson vaccine. KSMC is a tertiary center in Riyadh with approximately 6000 deliveries annually, according to KSMC statistics. This study demonstrated that hypertension-related diseases occurred more in the vaccinated group than in the unvaccinated group, and this result is comparable to that of a previously published study, which showed that the COVID-19 vaccine might play a role in acute blood pressure elevation due to an imbalance between angiotensin II (overactivity) and angiotensin [16 ]. Preeclampsia was noticed more frequently among vaccinated women than among unvaccinated ones. It was already known that COVID-19 causes pathophysiological changes that can cause preeclampsia during pregnancy [17]. Conversely, a recent study demonstrated that the COVID-19 vaccine does not cause or lead to preeclampsia [18]. It was noticed in this study that thyroid dysfunction was more common in the vaccinated group, irrespective of whether the diagnosis was made before pregnancy or recently discovered during pregnancy workup; however, none of them had a thyroid crisis or was admitted to the ICU. Some reports mentioned that the COVID-19 vaccine could induce some autoimmune or inflammatory adverse effects [19]. Even so, the benefits of the COVID-19 vaccine outweigh the risk of dysfunctional thyroid disorders [20]. Our study shows that the COVID-19 vaccine does not increase the rate of gestational diabetes. This result is similar to that of a previous report that established the safety of the COVID-19 vaccine and did not link it to an increased risk of gestational diabetes [21]. A systematic review could not prove any relationship between the COVID-19 vaccine and gestational diabetes [22]. This study showed that the COVID-19 vaccine caused neither polyhydramnios nor oligohydramnios. These findings are comparable to the results of a previous study [9]. This study showed more NICU admissions for NBs delivered to vaccinated women than for those born to unvaccinated women, and most of these admissions were due to low Apgar scores and respiratory complications. These findings were not linked to any type of vaccine or to whether the women received the vaccine before or during pregnancy. No neonatal deaths were reported. A previous observational study found that exposure to mRNA was not associated with higher adverse pregnancy or neonatal outcomes in terms of NICU admission [23]. Another published study investigating the relationship between COVID-19 vaccination in pregnancy and adverse perinatal outcomes showed no increase in the rate of NICU admission nor did it indicate low Apgar scores [24]. Another recent review did not show any relationship between COVID-19 vaccination and adverse perinatal outcomes [25]. Regarding the gestational age and birth weight in women who received the COVID-19 vaccine during pregnancy, our study revealed that the average gestational age of unvaccinated women was higher than that of vaccinated women. Evidence from prior research also supports this finding [26]. It was also noted that the mean gestational age of vaccinated women for the second and third doses was nearly identical to that of vaccinated women for the first dose. In contrast to this result, one study demonstrated that the gestational age of women who received a second dose of the COVID-19 vaccine was higher than that of women who had received only one dose [27]. Moreover, the average birth weight of NBs delivered by unvaccinated women was similar to that of NBs delivered by vaccinated women; however, the birth weight increased after the second dose and even more after the third dose. As confirmed by previously published studies, no difference in gestational age and birth weight was observed between vaccinated and unvaccinated pregnant women [27-30].

Strength

This study examines women who received the COVID-19 vaccine before or during pregnancy with all the available types of vaccine at the time of the study.

Limitations

It is a single-center study that lacks extended follow-up of NBs.

## Conclusions

At the start of the pandemic, safety data on COVID-19 immunization in pregnant women and perinatal outcomes were scarce. However, later evidence demonstrated that the administration of COVID-19 vaccines to pregnant women had no harmful maternal or neonatal consequences. Our findings add to the growing body of evidence supporting the safety of COVID-19 vaccination for pregnant women prior to, during, and after pregnancy. Our study confirmed that the COVID-19 vaccine, regardless of the type received, has no adverse perinatal outcomes for pregnant women or NBs. Moreover, the vaccines from Pfizer, Moderna, and AstraZeneca were all safe, with only minor changes in gestational age and birth weight.
